# Mechanisms at the core of the Chinese script invention

**DOI:** 10.1515/cog-2025-0041

**Published:** 2026-02-02

**Authors:** Ludovica Ottaviano, Silvia Ferrara

**Affiliations:** Department of Classical Philology and Italian Studies, 9296 University of Bologna, Bologna, Italy

**Keywords:** Chinese script, metaphor, metonymy, invention

## Abstract

This article explores the cognitive foundations of Chinese writing by analyzing the earliest attestations on oracle bone and bronze inscriptions from the Late Shang (1250–1045 BCE) to the Western Zhou (1045–771 BCE) periods. Integrating palaeographic and cognitive perspectives, we show that metaphorical and metonymic devices of the visual type, through visual emphasis on key configurations (such as *pars pro toto*), conceptual extension of meaning, and organization of iconic core components, were crucial in shaping and anchoring the visual and semiotic architecture of the script. These cognitive mechanisms reveal a primacy of the visual structure of the script, supported by neuroscientific evidence of direct orthography-to-semantics mapping, with phonology acting only as a secondary refining mechanism. These findings challenge traditional phonocentric models of writing evolution and illustrate an internal developmental trajectory grounded on visual metaphor and metonymy that confirms, at last, the independent invention of Chinese writing.

## 1 Iconicity at the core of early Chinese characters

Early writing systems, though often iconically grounded, tend to be analyzed through an evolutionary lens that prioritizes language encoding over visual communication (Daniels 1992; Sampson 1985; and others), frequently presenting graphic development as a progression toward compression and abstraction (Kelly et al. 2021).1Compression is a process by which graphic forms lose unnecessary graphic features over time as they are repeatedly remembered, reproduced, and transmitted from one individual to another. This streamlining occurs because individuals naturally simplify information for easier processing and transmission, conveying the same core meaning with less detail and effort (Garrod et al. 2007; Kelly et al. 2021; Tamariz and Kirby 2015). However, iconic representation was fundamental to the invention of writing across diverse cultures, from Mesopotamia and Egypt to China and Mesoamerica, as it served as an essential means for conveying layered meanings beyond language representation (Ferrara 2018; Houston 2004), and possessed mnemonic and immediate communicative potential (Déléage 2013). In the Chinese tradition, the functional role of iconicity was formally acknowledged as early as the second century CE in the ‘six script’ (*liushu* 六書) classification by Xu Shen 許慎 (58–148 CE). This theory has since been revisited and refined, with iconic signs recognized as representing words through semantographs (pictographs, deictic graphs, and quasi-pictorial graphs), rebus (loangraphs),2The rebus principle is the process by which an image represents syllables or words through homophony (e.g., a bee 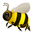 for ‘to be’). In traditional Chinese scholarship, this phenomenon is known as *jiajie* (phonetic borrowing or loangraph), whereby a sign originally depicting a kind of wheat 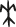 (*mə.rˤәk), for instance, was used to represent the verb ‘to come’ (*mə.rˤәk). Here and throughout this paper, unless otherwise specified, Old Chinese reconstructions follow the system of Baxter and Sagart (2014). and their integration into semantic and phonosemantic compounds (Bottéro 1998; Qiu 2000). Yet, the relationship between iconicity and visual inference – in terms of metaphor and metonymy – remains an under-explored dimension of early Chinese writing.3Recent Chinese scholarship has introduced structural approaches to character formation which offer valuable insight into the mechanisms of graphic creation, though not explicitely grounded in cognitive theory. According to this approach, character formation can be divided into original creation (which follows a mimetic mechanism drawing from real or imagined objects) and derivative creation (which modifies the original creations) (e.g., Deng 2005, 2008; Bai 2011). Original creations include partial representation, single and compound forms, and the use of additional elements, while derivative creation include deformation of basic characters (e.g., orientation and positioning, rotations), addition of non-character components to basic characters, and combination of basic characters to form new characters.


Neuroscientific studies in reading Chinese today demonstrate the importance of visual-semiotic structures (see, for example, Lin et al. 2011; Liu et al. 2003; Zhang et al. 2019), warranting an in-depth appraisal of how the visual shapes and composition of early Chinese characters enabled cognitive access to conceptual frameworks, anchoring visual lexical units (semantic and phonetic) and establishing the orthographic architecture of characters.

Our investigation focuses on the earliest attested forms of the Chinese script, which functioned as a fully developed writing system which registered an early stage of the language that later branched out into the various forms now known as Chinese: the oracle bone inscriptions (OBI, *jiaguwen* 甲骨文) from the Late Shang (ca. 1250–1045 BCE) and the bronze inscriptions (BI, *jinwen* 金文) from the Late Shang and the Western Zhou (1045–771 BCE) periods. These two primary media reflect the same system, albeit with stylistic differences. While these may not be the very earliest records of Chinese writing, limited archaeological evidence complicates the reconstruction of its earlier stages (Boltz 1994; Demattè 2022; Keightley 2006; Qiu 2000), making them the most reliable sources for any meaningful analysis.

Oracle bone inscriptions, carved on turtle plastrons and bovine scapulas, comprise over 160,000 inscribed pieces with 4,000 distinct signs (Li et al. 2020). These inscriptions evolved through simplification and increasing complexity across stylistic groups over five periods (Chen 2007b; Huang 2007; Qiu 2000). Bronze inscriptions, emerging in the Early Shang (ca. 1600 BCE) with concise, highly iconic clan emblems – likely early forms of writing (Xie 2022) – grew to longer texts by the Western Zhou, reaching up to 500 characters and becoming progressively linear and streamlined across three periods (Li 2018; Qiu 2000). With approximately 21,000 inscribed bronzes and 4,500 distinct signs, including 700 emblems, bronze inscriptions mark a pivotal phase in the development of Chinese writing (Dong 2011).

**Figure 1:**
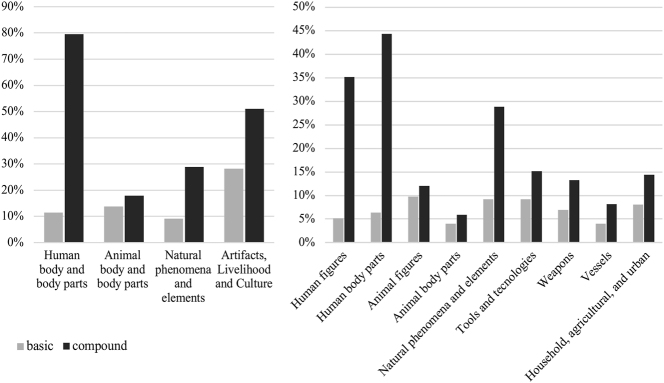
Distribution of iconic signs as basic self-standing signs and as modified or compound forms. The left panel presents comprehensive categories, while the right panel provides a more detailed breakdown of specific subcategories. Data are plotted to highlight the differences in frequency and usage patterns.

OBI and BI characters are primarily composed of iconic signs, while geometric and abstract shapes are also present. These basic graphic units can stand alone, be modified or form compound characters, which make up most of the Chinese repertoire. The OBI corpus contains only 174 basic units (OBC library),4This number is based on the classification of sign components from the OBC library, available at http://jgw.aynu.edu.cn/ (accessed 1 December 2024). Different counts include 164 by Shima (1971), 149 by Yao and Ding (1989), 150 by Yu (1996), 144 by Shen and Jinyan (2017), and 148 by Li (2012). just 5 % of the total sign inventory, highlighting a versatile system representing words5Here, we use the term “word” generically because early Chinese writing did not systematically encode all linguistic levels (phonological, morphological, and semantic) and that morphology was subordinate to larger linguistic units, suggesting that written signs functioned at a higher level of abstraction, closer to words than individual morphemes (Behr 2009). Moreover, Old Chinese words could be multi-morphemic and multi-syllabic yet represented by a single character (e.g., 鼻 ‘to smell,’ reconstructed as *Cə-bi[t]-s), reinforcing the word-based nature of the early script (Handel 2019). built on a small set of core components. Of these basic units, 63 % are iconic, a conservative estimate given that over half of the corpus remains undeciphered (Li et al. 2020). We can group these icons into four macro categories: (1) human body and body parts (e.g., 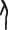, 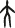, 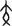, 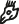, 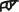, 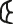), (2) animal body and body parts (e.g., 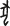, 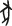, 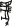, 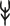, 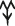, 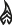), (3) natural phenomena and elements (e.g., 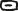, 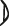, 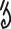, 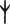, 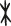, 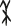), and (4) artifacts, livelihood, and culture (e.g., 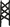, 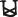, 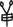, 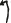, 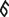, 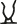). Uncertain cases, such as 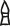, interpreted either as a phallus (Guo Moruo in Yu 1996: 3555) or, more plausibly, a sacrificial offering tray (Chen 2007a), are excluded to maintain analytical clarity. Icons appear either as self-standing simple characters or as components in modified or compound forms (Figure 1).

Human-agent components, though present in only 11.5 % of basic characters, appear in nearly 80 % of compound characters, emphasizing the crucial role of embodied cognition in representing abstract concepts (Kwan 2017; Lakoff and Johnson 1999). For instance, a ‘hand’ 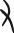, which physically holds objects, can extend metonymically to ‘obtaining’ in 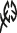 (a hand holding a seashell, used as currency, symbolizing the acquisition of wealth) and ‘ruler’ or ‘high-ranking minister’ in 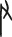 (a hand holding a stick, associated with authority). At the same time, the hand 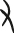 (*[ɢ]ʷəʔ) serves as rebus for ‘have, exist’ (有, *[ɢ]ʷəʔ) and ‘also, in addition’ (又, *[ɢ]ʷəʔ-s). In contrast, tools and cultural artifacts, which function more frequently as standalone characters (28 %), appear less often as components in compounds (51 %). This is likely due to their concrete, context-bound semantics: since they denote specific objects with limited metaphorical range, these graphs are less adaptable for recombination. As such, they encode object-centered affordances that resist abstraction, thus yielding fewer derivative compounds.

Iconicity thus facilitates key mechanisms for word notation: polysemy (also referred to as polyvalency or polyphony, where a single sign can represent multiple meanings through semantic association) and homonymy (rebus or paronomasia, which expresses phonetic similarity or sound proximity) (Behr 2009; Qiu 2000; but see Boltz 1994). While semantic association was the dominant strategy in the early formation of signs, a shift toward phoneticization is evident in the bronze inscriptions of the Zhou period, marked by the growing prevalence of phonosemantic compounds (Li 1993: 3; Huang 2003: 3). This systemic trend reflects an increasing integration of visual and phonetic cues within a single character rather than a full transition to phonograms, shaping the long-term development of Chinese writing.

Polysemy is intrinsically tied to the visual dimension of Chinese characters, from transparent iconicity to metaphor. Here we argue that specific cognitive devices, namely metaphor and metonymy, operated within the visual modality of the early semantographs and semantic compounds, and the visual-aural multimodality of phonosemantic compounds, being pivotal to the invention and development of Chinese writing.

Metaphor and metonymy are, indeed, fundamental mechanisms of conceptualization and meaning construction (Lakoff and Johnson 1980/2003; Gibbs 2008; Littlemore 2015). Recent scholarship has broadened this view to visual and multimodal domains, showing that these mechanisms are not limited to language but are grounded in general cognitive and perceptual capacities. Metaphor connects abstract concepts to familiar, concrete ones (e.g., ideas are light-sources, via the image of a lightbulb (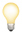) or expressions such as “brilliant ideas” and “to shed light on ideas”). Metonymy, a related sub-process, establishes contiguity within a domain (e.g., “crown” or 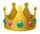 for monarchy). Studies in visual and multimodal metaphor and metonymy (e.g., Bolognesi 2017; Feng 2017; Forceville 1996, 2008; Forceville and Urios-Aparisi 2009; Kress and Van Leeuwen 2006; Pérez-Sobrino 2016) have demonstrated how images give tangible form to conceptual mappings and domain contiguities, often interacting in complex *metaphtonymic* relationships (Goossens 2003). In multimodal media such as in print advertisements, which combine image and text, the two modes interact for meaning making (Forceville 2008: 470), with visual features constraining interpretation (Bambini et al. 2024). This body of research provides a promising theoretical grounding for understanding how early Chinese graphs exploited perceptual salience and spatial organization, revealing the cognitive basis of the emergence and the development of the script.

We take a three-step examination. First, we investigate the strategies of salience that scribes used to select perceptually distinctive traits and design signs and abstract concepts. Salience, in scripts, can be visually exemplified by *pars pro toto* (the use of the most immediately accessible element as shorthand for an entire object or concept) and deictic metonymy (the manipulation of the visual context of the sign through the addition of non-iconic strokes to guide interpretation). Second, we examine how compound signs were formed, focusing on the role of visual cues to resolve ambiguities, to structure semantic relationships, and to expand the repertoire of signs. This reveals the cognitive principles at work. Third, we draw validation from a neuroscientific appraisal of visual, metalinguistic processing in contemporary Chinese reading, which strengthens our conclusion that early-stage iconicity is not merely a vestigial trait but an internal, functional affordance in the development of the script.

By explaining the formation of the early stages of Chinese writing purely through internal mechanisms of development, we can reconstruct the cognitive underpinning behind the creation of this script. This reconstruction indicates that the script emerged independently, without external models from other literate cultures and that metaphor and metonymy played a key role in script formation.

## 2 The metaphorical basis: visual and semantic building blocks of the Chinese script

The role of metaphor and metonymy in early writing is the focus of comparative investigations, where it was shown how visual metaphor reflects embodied human experiences (such as perspective, orientation, and body-container), which influenced scribal choices in organizing signs within two-dimensional spaces (Ottaviano et al. 2026). Also, two foundational principles, salience and categorization, further structure early scripts through visual metaphor and metonymy (Ferrara in print; Ottaviano et al. forthcoming). Salience refers to our visual attentional capacity for detecting diagnostic features and prominent graphic characteristics that facilitate the representation of complex ideas, as seen in the Chinese bow and arrow 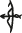 for ‘archer’ (object quality) or the proto-cuneiform ‘mouth’ 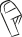 (deictic metonymy, with lines pointing to the mouth on a head). Categorization involves crafting prototypical icons and juxtaposing them with phonetic signs to organize knowledge both taxonomically, such as female figures for ‘women’ in Egyptian (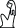) and Chinese (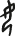), and metaphorically, such as a tilted down bull head 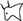 symbolizing ‘rage’ in Egyptian reflecting the conceptual metaphor anger is a dangerous animal. These principles reveal how cognitive and cultural factors are embedded into the design of early scripts. Building on these premises, we observe that early Chinese writing develops a unique system of compound signs, where visual cues were essential for both disambiguating existing signs and creating new ones. This tangibly sets it apart from other invented writing systems.

Studies addressing the metaphorical and metonymic bases of the Chinese script have mainly focused on isolated examples from the OBI corpus (Honkasalo 2011; Jiao 2023) or on specific phenomena such as “classifiers”6The term “classifiers” was initially and systematically adapted to Egyptian writing system (see Goldwasser 2002, 2023; Lincke and Kammerzell 2012; Kammerzell 2015) and extended to other complex writing systems. in later phonosemantic compounds (Chen 2016, 2024; Goldwasser and Handel 2024; Handel 2023; Xu 2024; Xu and Goldwasser 2024). Cognitive-semiotic analyses have clarified how iconicity, metonymy, and metaphor interact in the historical development of Chinese writing. Building on Peircean semiotics and cognitive linguistics, recent work has shown that non-phonetic graphs can be interpreted as icons, combined icons, and symbols, where many early characters operate through metonymic focusing (e.g., *jian* 見 ‘eye’ → ‘see’) or metaphorical extension (e.g., *peng* 朋 ‘paired shells’ → ‘friends’) (Liu and Tai 2016). The continuity between image, diagram, and metaphor has likewise been identified as a key mechanism for abstraction and conventionalization, reflecting the gradual transition from figurative to conceptual representation (Zhou 2021).

Graph-formation has further been analized using reference-point selection in the semantic organization of early Chinese writing: new graphs were systematically generated through transformations, additions, or truncations of existing signs, producing structured relations of categorization, metonymy, and metaphor between base and derivative forms (Deng and Chen 2018). Similar tendencies toward metaphorical organization are observed in investigations that reinterpret oracle bone and bronze inscriptions through the indexing components (often referred to as radicals) of the *Shuowen jiezi* 說文解字 (1st–2nd centuries CE) (Kwan 2011, 2017). However, such investigations rely on a later, systematized stage of the script where much original iconicity is compressed, abstracted, or lost. By contrast, our approach is bottom-up: it targets the earliest script sources and focuses on how metaphor and metonymy shaped the visual core components of the ancient characters and their combination into structured signs.

Selecting icons in the earliest stages of Chinese writing, as well as in all original invented scripts, is not just a cultural but also, more fundamentally, a cognitive process. Natural perspectives, visual system constraints, and object recognition processes that favour contours and patterns found in natural scenes (Changizi and Shimojo 2005; Dehaene 2010) are factors that guided early scribes in selecting and emphasizing specific, salient features of objects to maximize recognizability. Processes such as compression (Kelly et al. 2021) and simplification (Qiu 2000), evident as early as the OBI script, further contributed to the development of sign forms, with salience remaining central.

Salience governs attentional selection, as it enables the recognition of wholes through distinctive parts and diagnostic features – a process closely tied to *pars pro toto* metonymy (Langacker 1993; Lakoff 2008; Littlemore 2015; Ottaviano et al. forthcoming). This operates on a spectrum: a “light” *pars pro toto* retains contextual cues, as when tails distinguish pigs 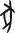 from dogs 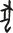 or horns and trunks differentiate rhinoceroses 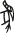 and elephants 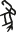 within a shared body frame, while a “true” *pars pro toto* abstracts a single feature, such as a caprid head 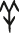 standing for the whole animal.

**Figure 2:**
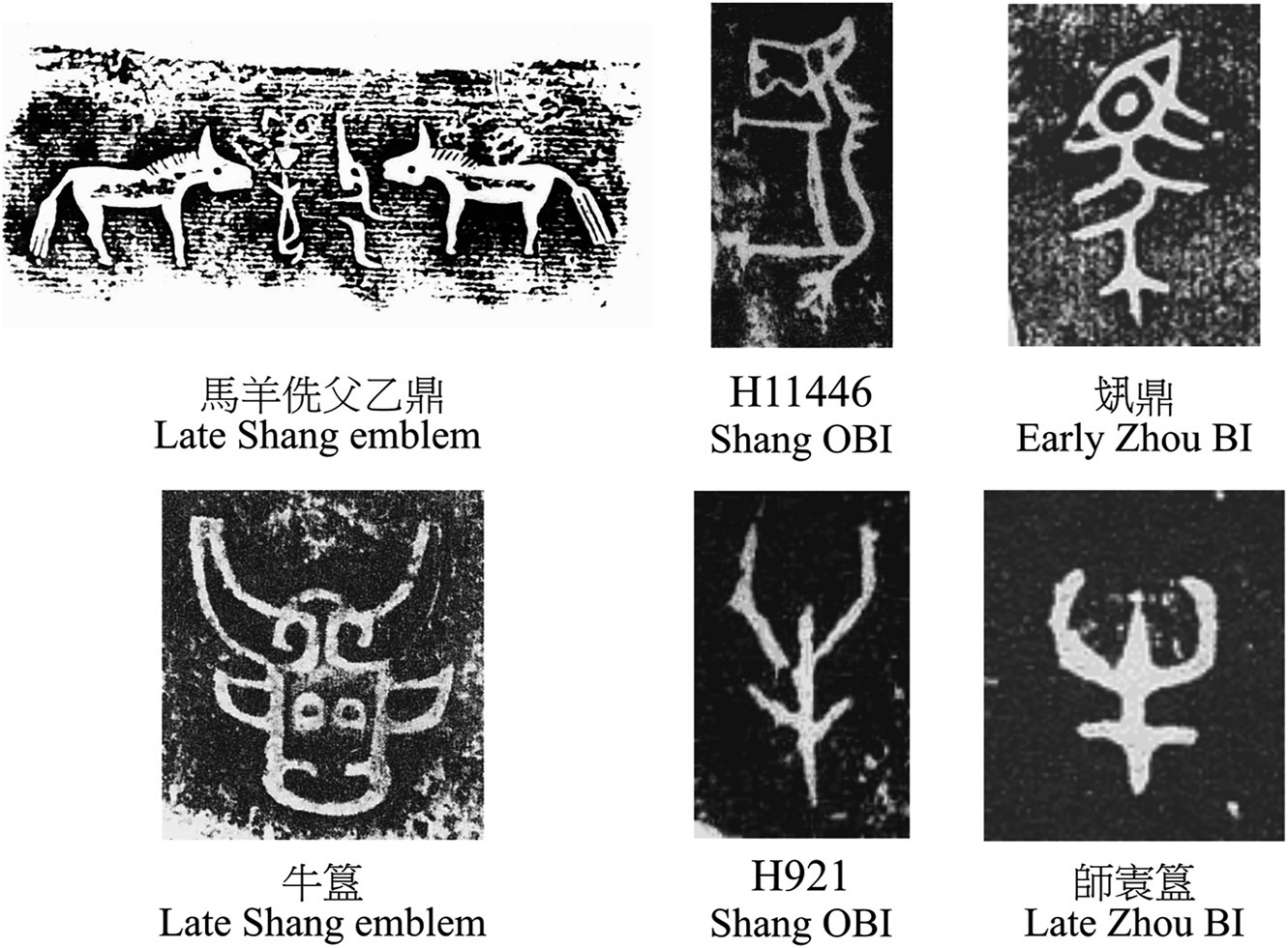
The horse (top) and the ox head (bottom).

Such selective representation stayed crucial even with later linearization and streamlining, which reduced, albeit not completely, the iconic basis of signs (Figure 2). For example, Shang bronze clan emblems depicted animals such as horses in highly pictorial styles that resembled real-world forms. However, in the OBI the sign was simplified to an outline and rotated 90° to accommodate vertical writing, but retained its most salient features: the mane and tail. The Zhou iteration became even more abstract and streamlined, yet its distinguishing features remained intact. Similarly, the “true” *pars pro toto* ox head was progressively compressed and streamlined, yet its curved horns remained, ensuring recognizability and preserving semantic function. This demonstrates how the script adapted, favouring compression and simplification without complete loss of iconic reference.

While animal figures often relied on a contextualized or decontextualized defining feature, human figures required a different approach. Scribes simplified figures by focusing on limb positioning to indicate body posture. A standing figure in profile 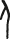 has loosely extended limbs facing one side, a front-facing figure 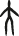 has symmetrical arms and legs, and a kneeling/sitting figure in profile 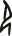 is distinguished by bent knees and resting arms on the thighs. These adjustments illustrate salience as a cognitive shortcut, maintaining clarity while minimizing unnecessary detail.

The introduction of new pictographs was rare (Huang 2003: 4). While most OBI iconic signs were retained in the Zhou BI script with minor modifications, a few pictographs were added to address evolving linguistic and cultural needs. For example, a new hand depiction 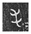, with five fingers, was designed to distinguish it from the OBI hand 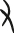 (three fingers), which had specific linguistic functions via rebus. Another example is the interlocking cheek teeth or molars in profile 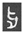, which replaced the front-facing teeth sign 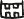, though the earlier form reappeared later, augmented by a phonetic element.

These examples illustrate how salience bridged visual representation and conceptual accessibility, occurring at a “basic level” of categorization in human cognition, balancing specificity with generality. Horns identify “ox”, while “animal” lacks this specificity. As idealized visual representations, icons functioned as visual “prototypes” (Hampton 2016; Lakoff and Johnson 1999; Lakoff and Johnson 2003; Rosch 1978), many of which could stand alone indicating physical, concrete objects (e.g., pig, person, ox). This inherent member-for-category quality allowed prototypes to participate in broader metonymic relationships (for linguistic parallels, see Littlemore 2015; Radden and Kovecses 1999), facilitating polysemy for abstract concepts (qualities, actions, events, groups, emotions). The stalk of grain 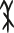 represents crops, the sun 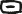 signifies both the celestial body and the ‘day’, and the moon 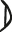 denotes both the celestial body as well as the concepts of ‘evening’ and ‘month’.

**Figure 3:**
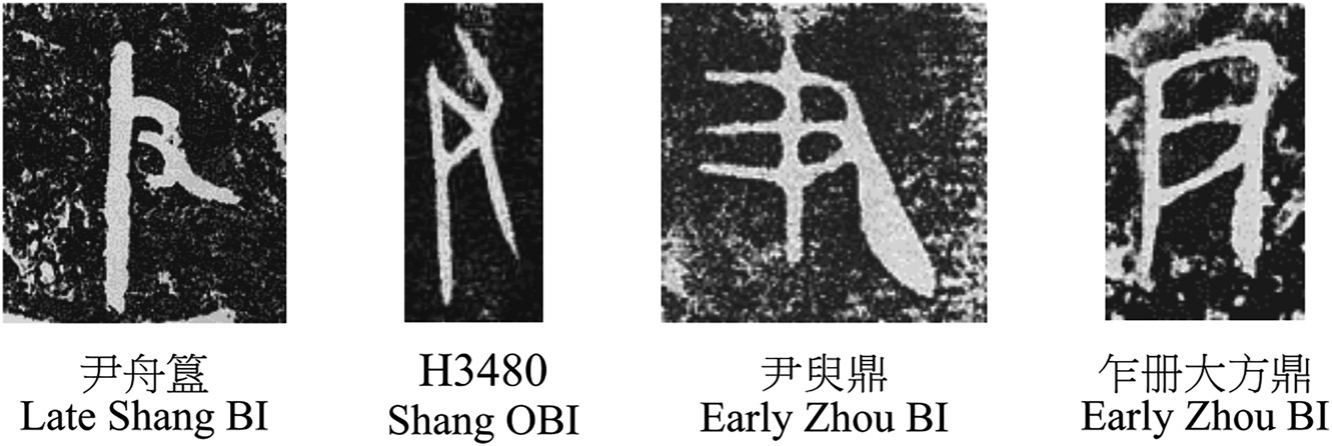
The hand-and-stick-ruler sign.

Notably, this strategy proved particularly effective for representing social roles through a distinctive object feature or quality. The horse sign (Figure 2, top), for example, represented both the animal and the official responsible for horses in the OBI and BI. Similarly, a hand-and-vertical-line, likely symbolizing a tool (a staff) associated with authority, referred to a ‘ruler’ or ‘high-ranking minister’ (Figure 3). While graphic changes occurred, as for the signs above, their semantic function remained stable.

**Figure 4:**
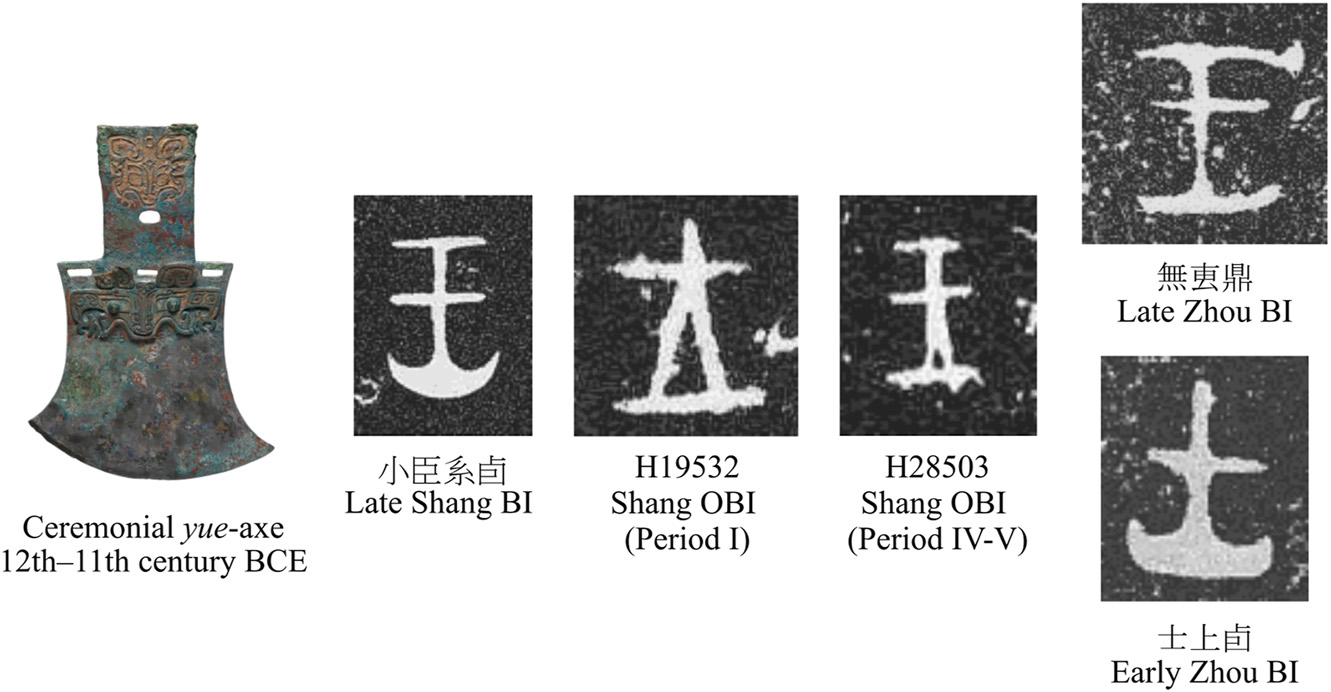
The graphic evolution of the king/warrior sign and the differentiation during the Zhou period. *Yue*-axe source: The Metropolitan Museum of Art, Gift of Ernest Erickson Foundation, 1985 (1985.214.24).

In contrast, the axe sign (a ceremonial *yue*-axe) offers a compelling example of how graphic alteration and societal demands influenced early scribes. Associated with clan leadership and military power, the *yue*-axe metonymically represented its wielder – a king or warrior. In the Shang BI, the sign was more pictorial, yet in the early OBI period, this iconicity persisted, though schematized, with the original curved blade rendered as a triangle (△), and later compressed into a ┴ shape (Figure 4). At that time, the character recorded two related words: ‘warrior’ (士, *[m-s-]rəʔ) and ‘leader, king’ (王, *ɢʷaŋ), reflecting the absence of a singular ‘king’ and the existence of multiple tribal leaders (Li 2020). By the late OBI period, a line was added on top, and during the Zhou period, it exclusively represented the king. The earlier form, retaining the curved blade, was repurposed to symbolize military soldiers and, more broadly, ‘men’. The hypothesis that the two characters shared a common origin in the *yue*-axe sign has long been supported (e.g., Xu 1934; Lin 1998), which pinpoints their gradual divergence towards semantic and visual specialization. This differentiation characterizes a rare expansion of the written visual vocabulary of the Zhou BI but at the same time shows the adaptability and flexibility of the script.

Another salient strategy is adding deictic symbols (circles or lines) to mark focal points (Qiu 2000: 183; Schwartz 2019). This deictic modification can be “literal”, as a hand with a circle attached to the arm 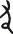 for ‘forearm’, or conceptual. OBI examples include dots in a woman’s breast (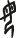) for 'mother', and a stroke or a square on top of a person (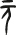 and 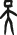) for the head as a metaphorical site, representing priority, beginnings, and ‘top’ (Jiao 2023: 43; Ottaviano et al. forthcoming). Specifically, 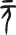 (profile figure with marking) meant ‘first’ and ‘beginning’, while 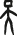 (front-facing figure with square) represented ‘top’. Later, the square streamlined and the sign took on the meaning of ‘sky, Heaven’ (Schwartz 2019). Deictic markers could also appear below signs, often defining ground-related actions (verbs), such as standing (e.g., a standing person on a line, 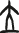) or sprouting and growing (e.g., grass on a line, 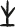). Both top and bottom lines reflected embodied up-down metaphors (Lakoff and Johnson 2003), bridging concrete representation with abstract and grammatical meanings.

Characters created through these strategies constitute a small percentage of deciphered OBI signs and an even a smaller proportion in BI, though some new signs did emerge during the Zhou period (Huang 2003). For instance, the ‘child’ sign with a deictic line on the head, 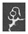/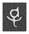, indicated the fontanelle, symbolizing ‘greatness’ by association with the profundity of caves or hollows (Schwartz 2019: 136). An alternative reading proposes that the line marks the breast, depicting a suckling infant and, by extension, denoting a “milk hole” (Ji 2014: 826–27). The sign for ‘sound’ 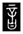, which first appeared in the Middle Zhou period (Jiang 2017: 91), added a dot inside the ‘mouth’ of the OBI ‘speech’ sign 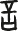, highlighting the source of sound. This ‘speech’ sign, in turn, is related to that of ‘tongue’ 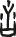 which differs only in the placement of the deictic lines. This progression – tongue and speech to sound – demonstrates how deictic markers shifted focus and meaning, moving from concrete to abstract.

The development of early iconic signs demonstrates a dynamic interplay between cognitive salience, metonymic representation, and script adaptation. While compression reduced pictorial details, the retention of salient features ensured recognizability and semantic stability. Though not the primary driver of sign expansion, salience was crucial to create the visual building blocks, anchoring the orthographic and semantic system of Chinese characters.

## 3 Grounding meaning: visual anchors in sign compounding

Building upon this core visual vocabulary, early Chinese writing expanded through two primary compounding processes: associating exclusively visual components (semantic compounds), and integrating visual and phonetic elements (phonosemantic compounds). We argue that both processes rely on visual relationships and a hierarchical organization of meaning.

**Figure 5:**
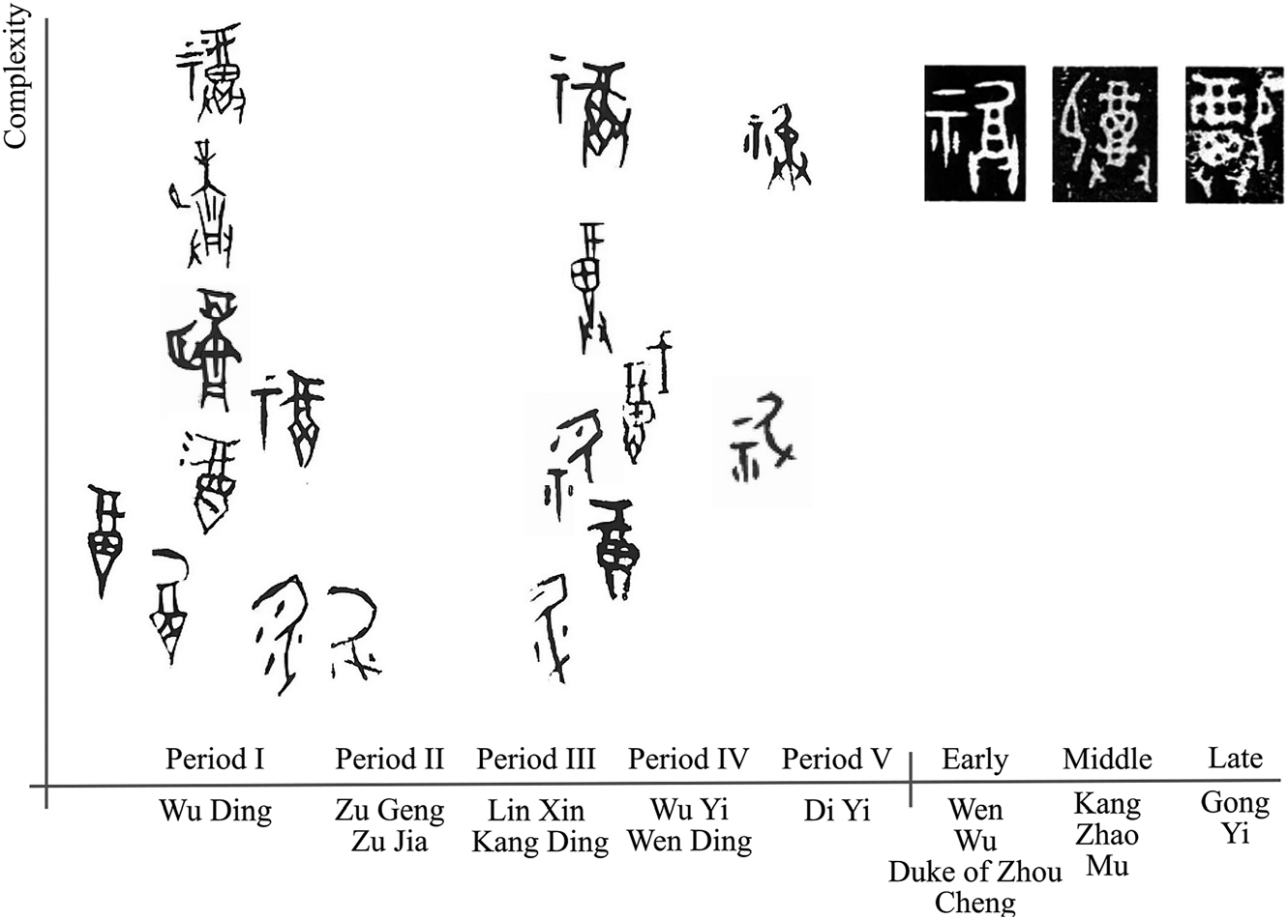
Disambiguating the sign for ‘libation’. The horizontal axis indicates time, and the vertical axis represents the level of complexity. OBI signs are collected from Liu (2014) and organized chronologically following Huang (2007); Zhou BI signs are sourced from Jiang (2017).

A prime example of semantic compounding for disambiguation is the large-mouthed wine vessel 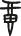, which resembled vessels unearthed at Early Shang sites. In the OBI, this sign metonymically represented both ‘wine’ (酉, *N-ruʔ) and the event ‘libation’ (祼, *[k]ˤor(ʔ)-s). It also served as a phonetic loan for 酉 (*N-ruʔ), an earthly branch. To disambiguate these meanings (or, as Qiu [2000: 226] puts it, to clarify the original meaning), scribes introduced visual adjustments making the sign for ‘libation’ a site of creative variation (Figure 5). Throughout the OBI period, the sign, not yet standardized, was depicted as a wine vessel with varying degrees of compression and simplification, often accompanied by dots (wine drops), a spirit tablet or deity, and/or two hands.7There was also an attempt, during a later period of the OBI, to pair the vessel with a dagger-axe 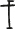 (戈, kwa<*kʷˤaj), which belongs to the same phonological series as ‘libation’ (祼, kwanH<*[k]ˤor(ʔ)-s) (Wang 2011). However, this variation is only sporadically attested and was quickly abandoned. This combination can be understood as a literal depiction of offering wine (with two hands) to a deity, though the abstraction from tablet to deity was already underway. Crucially, these visual additions established a hierarchy of semantic domains, prioritizing the ritual context.

Early Zhou scribes, adapting the OBI ‘libation’ signs, chose a complex version – vessel, tablet, hands – for their bronzes. By the Middle Zhou period, the tablet was replaced by a kneeling human figure, leveraging the metonymic association between posture and ceremonial kneeling. This adaptation, reflecting cultural priorities, firmly anchored the sign within the ritual domain, further emphasizing its dominance. This demonstrates that icons functioned as classifiers not only in phonosemantic but also in associative semantic compounds, mapping taxonomic-metonymic relationships and defining semantic domains.

Building on the principle of salience, associative compounds derived meaning not only through member-for-category taxonomy but also through other metonymic relationships based on events (action and causation) and qualities (perceptually or conceptually distinctive traits). Examples of categorization include the prototypical standing human figure in profile 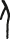 for the human domain, and the culturally contingent woman category, represented by a kneeling figure with folded hands before chest 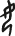, embodying qualities such as gentleness or submissiveness aligned with societal expectations of ancient China (Takashima 2020).

Action metonymies used human body parts (e.g., ‘foot’ 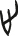) and/or urban elements (e.g., a *pars pro toto* ‘half-crossroads’ 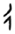) to represent movement. The sign 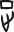, which combines a foot directed toward a target, conveyed ‘going toward a destination’ and was associated with activities such as inspections or hunting expeditions. A variant with the ‘crossroads’ classifier (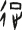 – 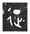), emerging as soon as the OBI, further refined the meaning to ‘campaigning’ or ‘military expedition’. Similarly, the ‘approaching’ sign (an inverted front view of a person 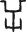) was combined with both foot and crossroads classifiers 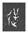 during the Zhou period, anchoring movement. Quality metonymies, instead, included animal attributes, such as the colour of the ox fur (via the *pars pro toto* ‘ox head’), to define qualities. In this way, signs 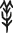 – 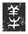 and 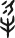 represented ‘reddish’, reflecting the association between ox hair and colour (Wang 1996), although this interpretation is still under discussion.

Spatial associations also played a distinct role. Though OBI signs lacked standardization (Keightley 1978; Qiu 2000), systematic trends reveal the use of orientation and positioning as deliberate strategies in script formation (Ottaviano et al. 2026). This approach leveraged embodied cognition and orientational metaphors (Lakoff and Johnson 2003), enabling visual iconic markers to define action and spatial relationships. For example, foot direction near a hill/mound determined meaning: upwards feet 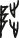 signified ‘to climb’, downwards feet 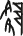 meant ‘to descend’. Similarly, human figures orientation shifted meaning: front-front 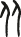 suggested ‘to follow’, while front-back 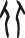 indicated ‘back-to-back’ or ‘the back’ (*pˤәk-s), eventually used as rebus to represent the ‘north’ (*pˤək). These modifications reflected the role of spatial differentiation in refining meaning, establishing relationships between sign components.

Rotations, too, were not merely aesthetic or practical adjustments, but also crucial visual cues for hierarchically organizing actions or states. An open mouth, for example, represented different actions depending on its orientation. Inverted and positioned above a food container (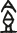 – 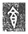) or a wine vessel (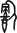 – 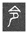), it indicated ‘eating’ or ‘drinking’. Above a kneeling/sitting person (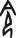 – 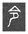), it conveyed ‘giving orders’, reflecting a top-down orientational metaphor. Rotated 90° and placed above a figure turning from a food vessel (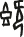 – 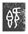), it meant ‘having finished eating’, hence ‘completed’ or ‘already’. These compounds exemplify the sophistication of early scribes in integrating visual logic and semantic precision.

The other key process, i.e. phonosemantic compounding, was also based on a hierarchical organization of semantic domains, where classifiers established broad conceptual categories, while phonetic elements refined meaning ensuring clear encoding of words. Two principal routes were followed for disambiguating signs or creating new ones: (1) adding classifiers to phonetic characters, or (2) adding phonetic elements to iconic signs (Qiu 2000: 221). While the systematic increment of phonosemantic compounds became more pronounced in later periods, these trends are already observable in the earliest stages of character formation.

The first route can be illustrated by the ‘wing’ pictograph 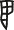 (翼, *ɢʷrәp). This sign was used as a loangraph for ‘the next day’ (翌, *ɢʷrәp) and was supplemented in the late OBI (periods IV and V) with the ‘sun’ classifier to disambiguate the sign, anchoring it within the time domain and resulting in the phonosemantic compound 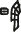. Examples of the second route include the early OBI phoenix pictograph 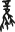.8The OC reconstruction, in this case, is taken from Schuessler (2009: 353) as the character 鳳 is not listed in Baxter and Sagart (2014). This sign is an iconic depiction of a bird with a tall crest and elaborate feathers, and was combined with the phonetic marker 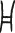 (*bam) in late OBI (periods IV and V), yielding forms such as 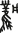 (鳳, *bəms). Another example is the ‘fur garment’ icon 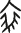, which depicted a coat with the fur side outward. In bronze inscriptions, this was modified by adding a hand 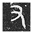 (*[ɢ]ʷəʔ-s) or, possibly, an insect with many feet 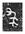 (*[g](r)u) as phonetic components, resulting in forms such as 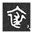 (*[g]ʷə). Additionally, the fur depiction was simplified to 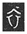, losing detail and becoming an iconic core component associated with clothing.

In summary, compounding processes demonstrate how early scribes organized meaning hierarchically, using visual strategies. By leveraging metonymic relationships and anchoring signs into semantic domains (e.g., ritual, movement, colour, time, and clothing), visual iconic cues constrained interpretation, resolved polysemy and homophony, and enhanced the communicative potential of the script. Even though phonosemantic compounds played a role in the development of the script, visual-semantic classifiers remained a crucial stepping stone since they preserved semantic clarity and adaptability to linguistic nuances. This suggests that phonological mediation operated alongside structured visual processing, rather than replacing it.

## 4 Neuroscience strengthens the visual metaphor affordance

Neuroscientific studies validate the importance of visual-semiotic structures in contemporary Mandarin Chinese reading, reinforcing our conclusions that visual metaphors gave foundation to visual lexical units (semantic and phonetic) and anchored the orthographic architecture of characters. Phonology, of course, is involved in reading across all writing systems, including Chinese, but it is now widely accepted that its activation and function vary depending on the orthographic structure (Perfetti et al. 1992, 2013). In alphabetic systems such as English, phonological activation occurs rapidly and in parallel with letter identification – a process known as the “cascade” model (Coltheart et al. 1993). In contrast, Chinese follows a “threshold” model, where phonological activation is contingent upon full orthographic character identification.

The extent to which phonological mediation is required for meaning retrieval in Chinese reading remains a subject of debate. An orthography-phonology-semantics mapping pathway (Perfetti and Tan 1998) has been challenged by evidence which supports a visual-semantic route, where meaning is directly retrieved from orthographic composition (Chen and Shu 2001). In their primed-naming experiments, Chen and Shu investigated the temporal activation of semantic and phonological processing in Chinese character recognition. Participants were presented with pairs of characters from Perfetti and Tan (1998) that were graphically (e.g., *cun* 村 ‘village’ and *cai* 材 ‘timber, material’), semantically (*ma* 媽 ‘mum’ and *mu* 母 ‘mother’), or phonologically (*jiang* 講 ‘to tell’ and *jiang* 獎 ‘award’) similar, or completely unrelated. In each trial, a prime character (stimulus) preceded a target, which participants were required to name aloud as quickly as possible to determine if semantic or phonological relationships influenced recognition. The results demonstrated strong and reliable semantic priming, as participants recognized a character faster when it was preceded by a related meaning-based prime. In contrast, phonological (homophonic) priming was weak or entirely absent when the prime was shown for a short duration, suggesting that phonology does not significantly facilitate immediate character recognition in Chinese.

Moreover, Chen and Shu’s findings on graphical similarity reveal that character identification relies on component-based processing. Indeed, when the prime and target were graphically similar but had different pronunciations and meanings, target naming was significantly slowed down. This indicates that structured components help activate potential lexical candidates, facilitating recognition when a component is strongly associated with a single character, but causing delays when multiple characters share the same component.

According to Perfetti et al. (2005), the inhibition effect, that is the filtering of distracting information, emerges at the phonological level, suggesting that component-based activation alone does not immediately lead to lexical interpretation until phonological processing is engaged. This is described in the Lexical Constituency Model, which posits word identification as the dynamic interaction of three constituents – orthographic, phonological, and semantic information. Unlike alphabetic scripts, where letters and letter clusters correspond to phonetic elements and therefore activate phonology pre-lexically, Chinese characters are recognized holistically, with components corresponding to embedded lexical units and phonological information functioning not as the primary access route to meaning but as a refining feature that constrains interpretation (Perfetti et al. 2005, 2013).

Event-related potential (ERP) studies, which use electroencephalography (EEG) to track neural responses with millisecond-level precision, further support that orthographic recognition precedes phonological activation and that structured component positioning is critical for early visual word processing (Lin et al. 2011; Liu et al. 2003; Zhang et al. 2019). For instance, Lin et al. (2011) examined the N170 response (peaking at 150–200 ms) by testing real characters, pseudo-characters (visually plausible, constructed from legitimate components in common positions but unpronounceable), false-characters (legitimate components in unusual positions), and random stroke combinations. Only real and pseudo-characters elicited the left-lateralized N170, indicating that phonology is not required for early character recognition. Crucially, orthographic regularity drove the N170 response: stimuli with components in conventional positions (real and pseudo-characters) elicited a strong N170, while false-characters behaved like random stroke combinations, suggesting that the characters are processed according to ordered, rather than arbitrary, component positioning. This demonstrates that orthography, in Chinese, is anchored to precise visual blocks and spatial configurations.

Beyond the N170 response, a positive ERP shift is observed at 200 ms (P200) after the onset of a character (Liu et al. 2003; Zhang et al. 2019). The P200 is recognized as a graphic-processing marker sensitive to component-level features, indexing selective attention and visual feature detection, including orientation and shape (Luck and Hillyard 1994; Zhang et al. 2019). This suggests that character recognition in Chinese is driven by salient, structured visual components, not individual strokes, relying on spatial configurations that enhance recognition through visual metaphor and positional regularity.

In addition, Zhang et al. (2019) used a picture-character matching task to investigate how orthographic and semantic factors affect processing. In each trial, participants saw a black-and-white line drawing of an object (e.g., a dog) together with a Chinese character. The stimuli were manipulated along two dimensions: orthographic similarity (whether the character shares components with the picture’s name; O+ = similar, O− = dissimilar) and semantic relatedness (whether the character’s meaning is related to the picture; S+ = related, S− = unrelated). For example, when presented with the picture of a dog (which in Mandarin Chinese is pronounced *gou* and written 狗), participants could see the following targets: *lang* 狼 (‘wolf’) as O+S+, *cai* 猜 (‘guess’) as O+S−, *shu* 鼠 (‘mouse’) as O−S+, and *mei* 眉 (‘eyebrow’) as O−S−. Results showed that orthographic similarity increased processing difficulty (slower response times), especially for semantically unrelated characters, indicating lexical competition. This supports the idea that orthographic information influences lexical-semantic retrieval, providing evidence for a direct orthography-to-semantics mapping. These results confirm that Chinese is a hierarchical visual system, where character components serve as cognitive anchors, facilitating structured orthographic access in the absence of, or prior to, full phonological mediation.

Taken together, this evidence points in the direction of a marginal role played by close phonological reading especially in the early stages of this script. Moreover, the neurological experiments corroborate the explanation that we are offering, namely that early-stage visual iconicity was not a relic of an archaic writing system but an active, functional, and efficient affordance that structured and supported later graphic developments. The foundational role of salience in shaping signs through layered *pars pro toto* and deictic metonymy ensured that recognizable visual prototypes anchored the visual-semiotic character of the script.

As seen in early compounding processes, classifiers systematically mapped meaning across domains, enhancing disambiguation and hierarchical organization, even as phonosemantic compounds expanded. Thus, visual metaphor and metonymy provided a stable, foundational basis that enabled the expansion of the inventory over time through structured and systematically ordered visual components. These components serve as priming reference points for processing and recognizing characters. This challenges traditional models that view iconicity as a transitional phase in writing system development and instead confirms that the Chinese script successfully sustains its visual foundation for cognitive reasons rather than cultural *habitus* (i.e. the reproduction of inherited scribal conventions), while at the same time accommodating its system to changing phonological and linguistic demands.

## 5 Toward validating an independent invention grounded in metaphor and metonymy

That Chinese writing is an original invention is generally accepted (but see Bottéro 2004), yet the mechanisms that explain it are rarely treated. Here, we have shown that an internal trajectory can be reconstructed, and that it points toward an independent, original invention. This can be explained by four key steps:

1. Visual iconicity, which functions as a cognitive affordance, structuring and supporting associative processes.
2. Cognitive mechanisms: metaphorical mapping across domains and metonymic contiguity within a domain, which facilitated conceptual extensions crucial to writing.
3. Systematic and conventional interpretability, a type of metalinguistic processing that remains neurally mappable today.
4. Finally, phonology enters the picture and closes the system, allowing for the formal fine-tuning of the writing system.

These processual steps provide a necessary account of the formation of Chinese writing.

To assess the validity of this model, it is essential to consider how each step could, in principle, be falsified. The first step, visual iconicity, would be challenged if evidence were found that external influences demonstrably shaped the formation of early signs. For instance, if structural parallels with another script, particularly in fundamental graphemic principles were identified, it would suggest some level of diffusion rather than independent innovation. However, paleographic evidence supports a distinct development, as the earliest attestations of Chinese writing show unique structural features that set it apart from coeval scripts. Also, as Daniels (1992) and Bagley (2004) have argued, the OBI retain a high degree of iconicity, whereas by the mid-second millennium BCE, Mesopotamian cuneiform had become highly abstract and predominantly syllabic. The notion that a script devoid of iconic content could have directly influenced a system rich in visual elements is, therefore, difficult to sustain.

Second, the cognitive potential enabled by metaphor and metonymy in facilitating polysemy would be falsified if early Chinese characters were limited to solely represent concrete objects or facilitate rebus mechanisms. Alternatively, discontinuity between visual representation and metaphorical extension, with signs representing words though arbitrary abstraction or external imposition, would also challenge our argument. However, our findings demonstrate an internally driven process, where polysemy naturally arises from metaphor and metonymy, anchoring abstract meanings to visual forms.

Third, if the structural organization of the script were borrowed from another writing system, the hypothesis of an internally developed metalinguistic system would require revision. If another script provided a direct model for how Chinese writing structured its characters, then external influence would be confirmed. However, neuroscientific studies of script processing and the historical development of character components indicate that these structuring principles emerged organically within the Chinese system, with no documented evidence of external transmission.

Finally, if phonological components were absent in early stages or introduced much later through external influence, the last step, that of phonology closing the system, would be questioned. Instead, early Chinese characters incorporated phonetic readings, including phonetic complements that disambiguated signs, supporting the argument that phonology developed internally rather than through external diffusion.

This model provides crucial empirical support for central claims in Cognitive Linguistics, namely that metaphor and metonymy are not confined to language but are fundamental cognitive mechanisms shaping communication across modalities. The evidence that these processes guided the emergence and development of Chinese writing extends the explanatory scope from speech to visual representation, showing how conceptual mappings and contiguities can materialize as graphic structures.

In this sense, the Chinese script provides rare material validation for a cognitive principle at the basis of its formation, and it offers the opportunity to ground abstract linguistic theory within archaeological and paleographical data, while at the same time offering precious and compelling evidence that supports an autonomous and internally-driven explanation for its emergence.
